# Exploring the Influence of Soil Types on the Mineral Profile of Honey: Implications for Geographical Origin Prediction

**DOI:** 10.3390/foods13132006

**Published:** 2024-06-25

**Authors:** Simona Schmidlová, Zdeňka Javůrková, Bohuslava Tremlová, Józef Hernik, Barbara Prus, Slavomír Marcinčák, Dana Marcinčáková, Pavel Štarha, Helena Čížková, Vojtěch Kružík, Zsanett Bodor, Csilla Benedek, Dalibor Titěra, Jana Boržíková, Matej Pospiech

**Affiliations:** 1 Department of Plant Origin Food Sciences, Faculty of Veterinary Hygiene and Ecology, University of Veterinary Sciences Brno, 612 42 Brno, Czech Republic; s.ljasovska@gmail.com (S.S.); javurkovaz@vfu.cz (Z.J.); tremlovab@vfu.cz (B.T.); 2 Department of Land Management and Landscape Architecture, Faculty of Environmental Engineering and Land Surveying, University of Agriculture in Krakow, 31-120 Krakow, Poland; jozef.hernik@urk.edu.pl (J.H.); barbara.prus@urk.edu.pl (B.P.); 3 Department of Food Hygiene, Technology and Safety, University of Veterinary Medicine and Pharmacy in Košice, 041 81 Košice, Slovakia; slavomir.marcincak@uvlf.sk; 4 Department of Pharmacology and Toxicology, University of Veterinary Medicine and Pharmacy in Košice, 041 81 Košice, Slovakia; dana.marcincakova@uvlf.sk; 5 Department of Computer Graphics and Geometry, Faculty of Mechanical Engineering, Brno University of Technology, 616 69 Brno, Czech Republic; starha@fme.vutbr.cz; 6 Department of Food Preservation, Faculty of Food and Biochemical Technology, University of Chemistry and Technology, 160 00 Prague, Czech Republic; helena.cizkova@vscht.cz (H.Č.); vojtech.kruzik@vscht.cz (V.K.); 7 Department of Dietetics and Nutritional Science, Faculty of Health Sciences, Semmelweis University, 1088 Budapest, Hungary; zsanett.bodor93@gmail.com (Z.B.); benedek.csilla.se.etk@gmail.com (C.B.); 8 Bee Research Institute, Maslovice-Dol 94, 252 66 Libcice nad Vltavou, Czech Republic; titera@beedol.cz; 9 State Veterinary and Food Institute Dolný Kubín, Veterinary and Food Institute Košice, Hlinková 1, 043 65 Košice, Slovakia; borzikova@svu-ke.sk

**Keywords:** traces elements, Czech beekeepers, sustainability, GIS

## Abstract

Honey contains a wide range of inorganic substances. Their content can be influenced, i.e., by the type of soil on which the bee pasture is located. As part of this study, the mineral profile of 32 samples of honey from hobby beekeepers from the Czech Republic wasevaluated and then compared with soil types in the vicinity of the beehive location. Pearson’s correlation coefficient was used to express the relationship between mineral substances and soil type. There was a high correlation between antroposol and Zn (R = 0.98), Pb (R = 0.96), then between ranker and Mn (0.95), then regosol and Al (R = 0.97) (*p* < 0.05). A high negative correlation was found between regosol and Mg (R = −0.97), Cr (R = −0.98) and between redzinas and Al (R = −0.97) (*p* < 0.05). Both positive and negative high correlations were confirmed for phaeozem. The CART method subsequently proved that the characteristic elements for individual soil types are B, Ca, Mg, Ni, and Mn. The soil types of cambisol, fluvisol, gleysol, anthrosol, and kastanozem had the closest relationship with the elements mentioned, and it can therefore be assumed that their occurrence indicates the presence of these soil types within the range of beehive location.

## 1. Introduction

Honey is very variable in its composition. In addition to basic substances such as sugars and water, honey also contains a diverse array of mineral components, including essential minerals and potentially toxic elements. The composition of honey is strongly influenced by natural and anthropogenic influences. Although mineral substances and potentially toxic elements subgroups are less significant components of honey by volume, they play a vital role in evaluating its quality [1,2].

It is essential that honey is free of potential contaminating substances. It is estimated that the honey bee forages on plants growing in an area from 7 to 28 km^2^, depending on their need for food and its availability [3]. Honey bees collect pollen as a source of amino acids, fats, minerals, proteins, starch, sterols and vitamins. A diverse selection of floral sources is required for a bee to get all her nutritional needs [4]. Honey bees interact with a variety of matrices that can be measured for contaminant accumulation, such as freshly collected pollen, honey, stored pollen, and beeswax. Honey composition is the result of many processes, is useful for gathering information about the environment, and can be a suitable bioindicator of environmental pollution [5,6].

The idea of using bees and honey in the field of the environment goes back to J. Svoboda (1961) and E. Crane (1984), who believed that bees could provide valuable data on the environmental impact the authors proved that potentially toxic elements such as Cd, Pb and metalloid As in bees and bee products correspond as indicators of environmental pollution [7,8]. In research from 1962, J. Svoboda’s team recorded an increase in the content of the radionuclide strontium 90 in the environment through the monitoring of bees—most likely as a result of nuclear testing. In the following years, bees were increasingly used to monitor environmental pollution by potentially toxic elements in geological and urban surveys [9,10]. As Leita et al. (1996) [11] suggested, a network of hives located next to polluted areas can provide data for monitoring heavy metal emissions from specific sources. Ruschioni et al. (2013) [12] also show that trends in metal contamination correlate with weather patterns and anthropogenic activities in the region where samples were obtained. Honey has nutritional, medicinal, and prophylactic properties, which are contributed to by its chemical components. The concentration of mineral compounds ranges from 0.1% to 1.0%. In comparison with nectar honeys, honeydew honeys are higher in minerals, resulting in higher electrolytic conductivity [13]. Also, the mineral content influences the color and taste of honeys. The higher the quantity of metals and the darker the color is, the stronger the taste they will have [14]. The mineral profile is dominated by potassium, followed by calcium, magnesium, sodium, sulfur, and phosphorus. Trace elements include iron, copper, zinc, and manganese [15,16]. The main mineral substances come mainly from soil and nectar-bearing plants but can also come from anthropogenic sources [17,18]. 

There are relations between the mineral profile of honey and a soil type [19,20]. According to the international soil classification system, soils are divided into different groups, especially by particle size, texture classes, and mineral composition [21,22]. 

The Czech Republic has a very diverse spectrum of soil types. The mountains are dominated by coniferous forests, under which podzol soils are formed. In the lowlands, which are a very warm region, chernozems are found. The occurrence of different types of soils is also influenced by altitude, slope, and biota. For example, alluvial soils, for the formation of which sufficient water is essential, are most often found near watercourses [23]. The Czech Geological Survey provides a detailed map of soil types, of which it is possible to evaluate the connection with the location of bee colonies. The mutual relationship between soil type and mineral substances in honey can be used to predict the geographical origin of honey. The aim of this study was to verify the influence of soil types according to the international classification on the mineral profile of honey depending on the total area of the soil type in the beehive location. A partial goal was to verify the correlation dependence of the mineral composition of honey and soil type and to describe the mineral profile depending on the soil type.

## 2. Materials and Methods

In this study, 32 multifloral honey samples were collected and harvested between 2019 and 2020 in the Czech Republic, Moravia. The honeys were collected from hobby beekeepers and harvested at the University of Veterinary Sciences with the same equipment to eliminate the impact of different harvesters. The pollen profile and locality are summarized in Table S1 and Figure S1. The quantitative melissopalynology analysis was performed with semiautomated acquisition according to the previous study [24]. 

The World Reference Base for Soil Resources (WRB) classification system [22] was used. The area of WRB for the beehive location was processed by QGIS 3.28 (QGIS Development Team, 2023); soil data were taken from the national geoportal https://geoportal.gov.cz/ (accessed on 1st April 2024) [25], where the soils are classified according to new soil systems [26].

In the collected data, 16 soil types were observed with different area sizes. The area and frequency of each soil type are detailed in Table 1. In general, hive locations were represented by more than one soil type, and the same soil type was observed in different hive locations. The evaluated area of soil type was represented by approximate bee flying distances of 3 km. In total, the 28.27 km^2^ for each hive location were evaluated. Honey samples were collected in situ by the research team directly from the hives. The GPS coordinates, land use data, and botanical profiles of the surrounding area were documented in a detailed questionnaire. The GPS coordinates of each hive served as the central point for a 3 km radius buffer zone. Within this defined buffer zone, soil-type data were extracted and analyzed.

The mineral content was determined by Inductively Coupled Plasma Mass Spectrometry ICP-MS 7900 (Agilent, Santa Clara, CA, USA) according to the STN EN 15763 [27], UNI EN 13805 [28], and UNI EN 13804 [29] in honey samples. The B, Na, Mg, Al, K, Ca, Cr, Mn, Fe, Ni, Cu, Zn, As, and Pb content were determined in each sample. The methods, including qualitative parameters, are described in Document S1.

The data were statistically evaluated by Xlstat 2024.2.0 (Adinsoft, Denver, CO, USA). The data follow normal distribution according to the Shapiro–Wilk test. For comparison of mineral content, ANOVA (post-hoc, Tukey HSD) and Pearson correlation coefficient were used. Due to the variation in soil types across different locations, statistical analyses were conducted using weighted correction methods. This approach adjusts for differences in sample size, ensuring that each observation contributes appropriately to the analysis. Weighted corrections were applied to ANOVA, Pearson correlation, and Classification and Regression Trees CART analyses. CART, based on a machine learning algorithm, was used to distinguish soil type based on mineral profile. The location of the hive positions was visualized in Excel 356 (Microsoft, Redmond, WA, USA).

## 3. Results and Discussion

The average mineral profile of Czech honey and its comparison with other European countries is shown in Table 2. The influence of the habitat of bees on the quality of their honey has been investigated in many studies [17]. Habitats influence the characteristic properties of honey, not only from the point of view of sensory uniqueness or the content of biologically active substances but also from the point of view of the mineral profile of the substances contained. The mineral composition of honey has been used in several studies, both for the characterization of bee honey [30,31,32,33,34] as well as a tool for proof of honey adulteration [33,35,36,37,38]. The mineral composition of honey is related to bee pasture [39] and is therefore significantly influenced by botanical taxa in the vicinity of the site [40] but also by geographic location and soil composition [41].

The measured values point to differences in the mineral composition of honey, which are related to both its botanical and geographical origin. However, it is clear from the comparison that K (1365.2 mg/kg) is the most represented element, followed by Ca (148.8 mg/kg), Na (36.9 mg/kg), and Mg (35.1 mg/kg). The greatest representation of K agrees with the results of other authors [33,35,39,41]. The representation of Ca and Na may differ depending on the country where the honey comes from when Italy (Latium region) and Turkey (Antolia) had a greater representation of Na [41,42]. In other studies, Ca was more represented, see Table 2, the same finding was confirmed in the Czech Republic (Moravia). All of the major mineral elements did not exceed the tolerable upper intake level (UI) for the adults, which are for Ca, Mg and Zn Fe, 2500, 250, and 25 mg/kg, respectively. For K, Mn, Mn, and Fe, there is not evidence in the EU for tolerable upper intake levels [43,44,45]. Considering the consumption of 1.7 kg [46] in the EU, honey is not a risk food, even in terms of potentially toxic elements.

**Table 2:** Comparison of the average mineral profile of honeys from different geographical areas.

	Present Study (Moravia Region) (*n* = 32)	Italy ^a^ (Siena) (*n* = 50)	Italy ^b^ (Latium Region) (*n* = 84)	Spain ^c^ (*n* = 40)	Spain ^d^ (Galicia) (*n* = 22)	Spain ^e^ (*n* = **)	Turkey ^f^ (Anatolia) (*n* = 30)	Ireland ^g^ (*n* = 50)	Portugal (Castelo Branco) ^h^ (*n* = 16)	Poland ^i^ (*n* = 30)	Hungary ^j^ (*n* = 34)
K (mg/kg)	1365.2	1195	472	1124	1345	1778	296	566	701.87	1585.6	610.2
Ca (mg/kg)	148.8	257	47.7	169	**	113	51	111	28.36	35.52	92.3
Na (mg/kg)	36.9	96.6	96	76	115	279	118	98	31.04	29	**
Mg (mg/kg)	35.1	56.7	37	39	77	136	33	31	74.00	**	17.6
Zn (mg/kg)	3.5	1.82	3.1	3.9	2.0	5.65	2.7	5	1.23	2.6	3.7
Mn (mg/kg)	2.3	1.54	3.0	3.4	5.2	**	1.0	4	2.78	2.72	2.1
Fe (mg/kg)	0.7	3.07	4.5	**	3.7	9.19	6.6	8	0.97	3.8	1.4
B (mg/kg)	11.1	**	**	5.43	**	**	**	**	**	5.17	**

** Not provided, ^a^ [41]; ^b^ [34]; ^c^ [35]; ^d^ [47]; ^e^ [29]; ^f^ [42]; ^g^ [48]; ^h^ [49]; ^i^ [50]; ^j^ [51].

As already mentioned, there can be more reasons for the different representations of mineral substances. Our study has shown that one of the factors influencing the mineral composition, specifically the content of K, Mg, and Mn, is the type of soil on which the colonies are located. Mineral representation in plants depends on the type of soil and the density of the root system, the amount of precipitation, and the mineral composition of the subsoil [52]. The average values of mineral substances in honey with respect to the soil types of the observed beehive location are indicated in Table 3. The most K was found in honey with a majority of podzol; on the contrary, the lowest amount was found in honeys from phaeozem, chernozem, and pseoudogley (*p* < 0.05). Higher K values in some types of soils can be explained by the fertilization of these soils [53]. Podzol soils also yielded higher amounts of Mg in honey (*p* < 0.05). High amounts of Ca were found in the honeys around the gleysols and rankers, but no statistically significant difference was found between the Ca content in the soils.

For B, Al, Ca, Cr, Mn, Fe, Ni, Cu, Zn, As, and Pb, statistical differences in the content of mineral substances in honey and the types of soil were not confirmed. This finding is due to the large variability of the measured values, while differences in the mineral composition of individual soil types were observed (Table 3 and Table 4), especially for B, Al, Mn, and Zn (*p* > 0.05).

The minor mineral substances such as As, Cr, Cu, Fe, Ni, and Pb are summarized in Table 4. Potentially toxic elements such as Pb and metalloid As can contaminate honey due to environmental pollution and Al. Higher concentration in Al in comparison with Pb and As was also confirmed in the Hungarian study, but the total amount of Al was lower than was detected in our study [51]. In our study, statistical differences between soil types and Pb and As were not determined. Cu, Fe, Ni, and Zn are essential nutrients for organisms, including bees and plants. Statistical differences with soil type have not been confirmed (Table 4). Low concentrations of minor mineral substances in honey were also confirmed in other studies [51,54].

In order to better express the relationship between mineral substances and soil types, the Pearson correlation coefficient was further used. The correlation between soil type and mineral substances is shown in Table 5. There was a high correlation between antroposol area and Zn (R = 0.98), Pb (R = 0.96), then between ranker area and Mn (0.95), then regosol area and Al (R = 0.97) (*p* < 0.05). A high negative correlation was between regosol area and Mg (R = −0.97), Cr (R = −0.98) and between the redzinas area and Al (R = −0.97) (*p* < 0.05).

A positive and negative high correlation was also confirmed for phaeozem, but this result is compromised by an error, which is due to the small representation of this soil in the analyzed localities, both in terms of total representation (1%) and frequency (number of occurrences: 2) (Table 5). At the same time, the frequency corresponded to two beehive locations where the phaeozem represented 20% and 10% of the given locality. Further research is still needed to define a conclusion for this type of soil so that it is included in more locations in a wider representation.

The relationship between mineral composition and plants has been confirmed in various studies, mostly focusing on plant mass, leaves, seeds, and roots [55,56,57,58]. Several studies [59,60] confirmed the effect of soil type on the nectar production of the nectar-bearing plant called mānuka (*Leptospermum scoparium*). Ca, Mn, and Fe contained in soil types had a positive effect on production. The amount of Ca also affects the number of flowers on plants [61,62]. The influence of soil type on the growth of other honey plants (*Salix caprea* and *Prunus padus*) was confirmed by [61]. On another honey plant, *Allium ursinum*, the influence of soil mineral composition on nectar production was also confirmed, where the influence of phosphorus was confirmed. The influence of humus, K, Fe, and Mn on the number of flowers was confirmed, while Mn also had an influence on the total nectar content. From the above, we expected that the effect on the mineral substances in honey is manifested due to higher nectar-producing capacity and the number of flowers on soils with a suitable mineral composition and a layer of humus. In our study, statistical differences between the type of soil and the content of Ca, Fe, and Mn in honey were not confirmed (Table 3), but the correlation dependence with the type of soil was confirmed (Table 5).

Therefore, methods of higher statistics were applied to verify the relationship between soil type and mineral composition, which allows for the comparison of several variable parameters. Classification and Regression Trees (CART) were utilized. CART is a machine learning algorithm that recursively splits the dataset based on features to predict a target variable (response). It constructs a decision tree suitable for classification, where the target variable represents categories or classes. In regression, the target variable represents a continuous variable. The CART reaches the best correct classification rate in comparison with not supervised (PCA) and supervised (LDA and QDA) classification for mineral substances [36] and for other honey parameters [63].

According to the CART, the B, Ca, Mg, Ni, and Mn in honey samples are characteristic of all soil types in our study. The soil types of cambisol, fluvisol, gleysol, anthrosol, and kastanozem were most closely related to the above-mentioned mineral substances found in honey and can, therefore, be assumed to have a major influence on the mineral content of honey (Table 6). An overall summary of the classification is provided in Figure S2. Cambisol and fuvisol are the most common soils in the Czech Republic. While cambisols are represented both in hilly areas and uplands and in mountains; fuvisols, on the other hand, were formed mainly in lowlands, especially along larger rivers [64]. In the Czech Republic, 58% of agricultural land is of the cambisol type [65]. These soils are poor in minerals; thus, in order to achieve adequate production, crops grown on them must be regularly fertilized, which affects their mineral profile [66]. According to CART, therefore, honeys with a large proportion of cambisol were mainly represented by a low content of B, Ca, and Mg, with the exception of the Ni content in honey, which increased with a larger area of cambisol in the vicinity of bee colonies.

The presence of fluvisol in the vicinity of the beehive location was manifested by a low content of B, Ca, and Mg in honey (29.4% of cases) or a low content of B and Ca (41.8% of cases) but in some cases, the presence of this type of soil led to a high content of B (6.5% of cases). These differences are explained by the type of soil, where fluvisol represents river sediments that can be affected by anthropogenic activity [67]. This fact is also indicated by the high content of Pb (Table 3), although in the discrimination according to CART, Pb was not significant, which is caused by the variability of this factor. Another type of soil that has been confirmed to have an effect on the mineral composition of honey is anthrosol. This type of soil is significantly transformed by human activity, mostly with originally less fertile soil [68], which, within the CART discrimination, was manifested by a higher representation of Ca and Mg. A high content of Ca and Mg is typical for anthrosol, while their higher content is due to both anthropogenic activity and sandy or sandstone subsoil [68,69]. Another type of soil influencing the mineral profile of honey, according to CART, was kastanozem. This type of soil is typical for pastures, steppes, meadows, and anthropogenic analogs [70]. Kastanozem was manifested by a high content of B and a low content of Ni. These soils are characterized by available Ca, Mg, and Na cations. In our study, only a higher Na content (Table 3) was confirmed in honey in relation to the amount of kastanozem in the location of the bee colonies. The content of B and Ni can be affected by anthropogenic activity (fertilization), but there is not enough information in the scientific literature about its content and availability for plants. Pollution as the reason for their higher content cannot be assumed because other metals such as Pb, As, Cu, and Zn have not been confirmed in honey.

## 4. Conclusions

The mineral profile of honey can be influenced, among mechanisms, by the type of soil on which the beehive is located and which occurs within its flying range. In this study, positive high correlations were confirmed with certain soil types and specific elements, namely phaeozem with Na and K, as well as ranker with Mn, regosol with Al, and anthrosol with Zn and Pb, while a negative correlation between phaeozem with B, Mg, Al, Ca, Cr, Mn, Fe, Ni, Zn, regosol with Mg, Cr, and rendzinas with Al (*p* < 0.05). The higher statistics methods subsequently proved that some elements are characteristic of the given soil type. Using CART analysis, the linear regression dependence between Ca, B, Mg, and Mn and the cambisol, anthrosol, fluvisol, and kastanozem soils was confirmed. The mutual relationship between soil type and mineral substances in honey can be used to predict the geographical origin of honey. When working with national map data, soil profiles can be used to predict the mineral profile of honey using minerals such as B, Ca, Mg, Ni, and Mn and subsequently authenticate its geographical origin. 

## Figures and Tables

**Table 1:** Soil type frequency and area.

Categories	Frequencies	Lands Area (km^2^)	%
Anthrosol	8	24.147	2.669
Cambisol	26	427.149	47.218
Chernozem	7	92.438	10.218
Fluvisol	26	95.405	10.546
Gleysol	21	36.620	4.048
Kastanozem	14	66.888	7.394
Luvisol	13	52.301	5.781
Pararendzina	6	20.171	2.230
Pelozem	6	31.849	3.521
Phaeozem	2	8.905	0.984
Podzol	2	10.307	1.139
Pseudogley	15	20.158	2.228
Ranker	5	1.242	0.137
Regosol	7	11.010	1.217
Rendzinas	4	2.663	0.294
Water Bodies	8	3.388	0.375

**Table 3:** Comparison of the major mineral substance profiles of soil types in honey (mg/kg).

Soil	Al	B	Ca	K	Mg	Mn	Na	Zn
Gleysol	392.2 ^a^	9.7 ^a^	196.9 ^a^	1604.9 ^abcd^	34.1 ^ab^	1.8 ^a^	41.7 ^ab^	3.8 ^a^
Cambisol	513.1 ^a^	10.3 ^a^	167.7 ^a^	1463.3 ^abcd^	34.7 ^ab^	2.8 ^a^	35.6 ^b^	4.1 ^a^
Luvisol	431.8 ^a^	10.6 ^a^	163.9 ^a^	1559.6 ^abcd^	32.3 ^ab^	1.9 ^a^	42.8 ^ab^	3.7 ^a^
Anthrosol	20.6 ^a^	14.7 ^a^	161.3 ^a^	1427.8 ^abcd^	35.9± ^ab^	1.1 ^a^	36.4 ^b^	6.3 ^a^
Podzol	11 ^a^	14.1 ^a^	174.7 ^a^	2099 ^a^	45.7 ^a^	2.8 ^a^	32.7 ^b^	2.9 ^a^
Pelozem	25.3 ^a^	14.7 ^a^	163.9 ^a^	1844.6 ^abc^	43.7 ^ab^	2.5 ^a^	32.4 ^b^	2.9 ^a^
Rendzinas	49.6 ^a^	9.6 ^a^	152.5 ^a^	1884.6 ^ab^	33.4 ^ab^	4.1 ^a^	31.8 ^b^	5.3 ^a^
Fluvisol	318.1 ^a^	10.7 ^a^	129.5 ^a^	1398.6 ^abcd^	33.9 ^ab^	2.5 ^a^	35.5 ^b^	2.9 ^a^
Ranker	144.6 ^a^	11 ^a^	189.2 ^a^	1272.6 ^abcd^	38.3 ^ab^	1.5 ^a^	33.2 ^b^	2.8 ^a^
Kastanozem	553.4 ^a^	11.2 ^a^	127.6 ^a^	963.7 ^abcd^	29.2 ^ab^	1.7 ^a^	35.5 ^b^	3.6 ^a^
Chernozem	318.2 ^a^	11.7 ^a^	125.3 ^a^	665.1 ^cd^	28.6 ^ab^	0.5 ^a^	45.1 ^ab^	3.4 ^a^
Pseudogley	54.5 ^a^	15.1 ^a^	119.2 ^a^	666.6 ^cd^	29 ^ab^	1.4 ^a^	32.6 ^b^	3.1 ^a^
Pararendzina	13.1 ^a^	13.7 ^a^	115.3 ^a^	878.7 ^bcd^	32.7 ^ab^	1.7 ^a^	25.1 ^b^	3.6 ^a^
Regosol	71.9 ^a^	15.7 ^a^	133.5 ^a^	985.6 ^abcd^	30.2 ^ab^	1.2 ^a^	28.4 ^b^	2.7 ^a^
Phaeozem	31.9 ^a^	9.7 ^a^	96.1 ^a^	477.7 ^d^	26.3 ^ab^	0.3 ^a^	64 ^a^	1.7 ^a^
Water Bodies	36.6 ^a^	10.7 ^a^	109.7 ^a^	916.6 ^abcd^	24.5 ^b^	1.8 ^a^	26.5 ^b^	2.3 ^a^

Different letters mean significant differences between raw (*p* < 0.05).

**Table 4:** Comparison of the minor mineral substances profiles of soil types in honey (mg/kg).

Soil	As	Cr	Cu	Fe	Ni	Pb
Gleysol	0.008	0.151	0.302	0.787	0.211	0.090
Cambisol	0.006	0.131	0.342	0.717	0.224	0.100
Luvisol	0.007	0.141	0.299	0.796	0.200	0.095
Anthrosol	0.008	0.151	0.370	0.749	0.173	0.086
Podzol	0.009	0.130	0.387	0.576	0.261	0.059
Pelozem	0.009	0.121	0.361	0.562	0.249	0.060
Rendzinas	0.008	0.130	0.366	0.591	0.184	0.072
Fluvisol	0.007	0.111	0.322	0.662	0.165	0.082
Ranker	0.005	0.128	0.425	0.621	0.144	0.066
Kastanozem	0.008	0.109	0.264	0.863	0.148	0.067
Chernozem	0.006	0.113	0.307	0.698	0.126	0.074
Pseudogley	0.005	0.132	0.183	0.562	0.211	0.048
Pararendzina	0.005	0.119	0.223	0.606	0.201	0.070
Regosol	0.006	0.092	0.249	0.502	0.165	0.052
Phaeozem	0.007	0.120	0.256	0.543	0.107	0.055
Water Bodies	0.006	0.100	0.172	0.481	0.127	0.043

Note: As, Cr, Cu, Fe, Ni, and Pb were no significant differences between raw (*p* < 0.05).

**Table 5:** Correlation between the area of soil type and mineral content in honey in the observed localities.

	B	Na	Mg	Al	K	Ca	Cr	Mn	Fe	Ni	Cu	Zn	As	Pb	No. of Locality
Anthrosol	**0.712**	**0.240**	−0.144	0.045	**0.344**	**0.742**	**0.595**	**−0.227**	**0.824**	−0.054	**0.812**	**0.982**	**0.375**	**0.956**	8
Cambisol	−0.013	0.037	−0.034	0.041	**0.087**	**0.243**	**0.191**	0.018	0.031	**0.290**	0.001	**0.263**	**0.087**	−0.027	26
Chernozem	**0.134**	**−0.530**	**−0.176**	**−0.263**	**−0.457**	−0.063	**−0.486**	**−0.370**	**−0.371**	**−0.437**	**−0.291**	**−0.349**	**−0.717**	**−0.357**	7
Fluvisol	**0.329**	**−0.140**	**−0.129**	**−0.139**	**−0.251**	**−0.513**	**−0.387**	**−0.192**	**−0.181**	**−0.337**	**−0.287**	**−0.356**	0.026	**−0.139**	26
Gleysol	**−0.674**	**0.519**	**−0.343**	−0.163	**0.344**	**0.296**	**0.574**	**−0.457**	**0.237**	**−0.372**	**−0.383**	−0.166	**0.489**	−0.172	21
Kastanozem	**−0.510**	0.001	**−0.594**	**−0.169**	**−0.339**	**−0.479**	**−0.215**	−0.077	**−0.199**	**−0.452**	**−0.444**	**−0.301**	**0.140**	**−0.375**	14
Luvisol	**−0.187**	0.103	−0.037	**−0.319**	**0.405**	**0.753**	**0.573**	**−0.489**	−0.148	−0.035	**−0.328**	**0.159**	**0.267**	**−0.266**	13
Pararendzina	**0.585**	**−0.333**	0.180	**−0.636**	**−0.822**	**−0.635**	0.007	**−0.517**	**0.293**	0.196	**−0.569**	**−0.294**	**−0.669**	−0.081	6
Pelozem	**−0.210**	−0.158	−0.057	**−0.306**	**0.218**	**0.285**	**0.259**	0.138	0.027	−0.062	0.050	**−0.239**	0.026	**−0.285**	6
Phaeozem	**−0.978**	**0.978**	**−0.978**	**−0.978**	**0.978**	**−0.978**	**−0.978**	**−0.978**	**−0.978**	**−0.978**	**0.978**	**−0.978**	**0.978**	**0.978**	2
Podzol	0.000	0.000	0.000	0.000	0.000	0.000	0.000	0.000	0.000	0.000	0.000	0.000	0.000	0.000	2
Pseudogley	**0.458**	**−0.440**	**−0.503**	**−0.448**	**−0.530**	**−0.374**	−0.238	**−0.534**	0.047	**−0.539**	**−0.583**	**−0.520**	−0.178	**−0.761**	15
Ranker	−0.247	0.235	0.687	−0.559	0.709	0.818	0.713	**0.954**	0.288	0.169	0.598	−0.386	0.450	−0.235	5
Regosol	**0.629**	**−0.741**	**−0.971**	**0.973**	**−0.946**	**−0.943**	**−0.982**	**−0.723**	**−0.886**	**−0.930**	**−0.906**	**−0.638**	**−0.568**	**−0.588**	7
Rendzinas	0.165	0.290	**0.796**	**−0.970**	**0.809**	0.485	**0.704**	0.139	0.304	0.471	0.629	0.375	**0.929**	0.387	4
Water Bodies	−0.107	−0.233	**−0.932**	−0.046	−0.366	−0.431	−0.085	−0.484	−0.182	**−0.698**	**−0.684**	−0.242	0.512	−0.560	8

Value in bold means significant correlation (*p* < 0.05); dark grey means positive correlation higher than 95%; light grey higher than 90%; dark blue negative means correlation higher than 95%; and light blue higher than 90%.

**Table 6:** Regression classification rules for mineral substances.

Nodes	Soil (Prediction)	Rules
Node 1	Cambisol	All cases
Node 2	Cambisol	If B ≤ 14.59 then Soil = Cambisol in 62.9% of cases
Node 3	Cambisol	If B (14.59; 15.87] then Soil = Cambisol in 8.8% of cases
Node 4	Cambisol	If B (15.87; 16.49] then Soil = Cambisol in 10% of cases
Node 5	Gleysol	If B (16.49; 17.05] then Soil = Gleysol in 11.8% of cases
Node 6	Fluvisol	If B > 17.05 then Soil = Fluvisol in 6.5% of cases
Node 7	Fluvisol	If B ≤ 14.59 and Ca ≤ 162.10 then Soil = Fluvisol in 41.8% of cases
Node 8	Cambisol	If B ≤ 14.59 and Ca (162.10; 179.20] then Soil = Cambisol in 5.3% of cases
Node 9	Cambisol	If B ≤ 14.59 and Ca (179.20; 189.20] then Soil = Cambisol in 5.3% of cases
Node 10	Anthrosol	If B ≤ 14.59 and Ca (189.20; 215.30] then Soil = Anthrosol in 2.4% of cases
Node 11	Cambisol	If B ≤ 14.59 and Ca > 215.30 then Soil = Cambisol in 8.2% of cases
Node 12	Cambisol	If B (14.59; 15.87] and Mg ≤ 30.22 then Soil = Cambisol in 4.1% of cases
Node 13	Anthrosol	If B (14.59; 15.87] and Mg > 30.22 then Soil = Anthrosol in 4.7% of cases
Node 14	Cambisol	If B (15.87; 16.49] and Ca ≤ 109.60 then Soil = Cambisol in 8.2% of cases
Node 15	Cambisol	If B (15.87; 16.49] and Ca > 109.60 then Soil = Cambisol in 1.8% of cases
Node 16	Kastanozem	If B (16.49; 17.05] and Ni ≤ 0.13 then Soil = Kastanozem in 5.9% of cases
Node 17	Cambisol	If B (16.49; 17.05] and Ni (0.13; 0.27] then Soil = Cambisol in 4.1% of cases
Node 18	Cambisol	If B (16.49; 17.05] and Ni > 0.27 then Soil = Cambisol in 1.8% of cases
Node 19	Anthrosol	If B > 17.05 and Mg ≤ 34.69 then Soil = Anthrosol in 4.7% of cases
Node 20	Fluvisol	If B > 17.05 and Mg > 34.69 then Soil = Fluvisol in 1.8% of cases
Node 21	Fluvisol	If B ≤ 14.59 and Ca ≤ 162.10 and Mg ≤ 31.49 then Soil = Fluvisol in 29.4% of cases
Node 22	Cambisol	If B ≤ 14.59 and Ca ≤ 162.10 and Mg (31.49; 37.11] then Soil = Cambisol in 4.1% of cases
Node 23	Cambisol	If B ≤ 14.59 and Ca ≤ 162.10 and Mg (37.11; 42.41] then Soil = Cambisol in 4.1% of cases
Node 24	Anthrosol	If B ≤ 14.59 and Ca ≤ 162.10 and Mg > 42.41 then Soil = Anthrosol in 4.1% of cases
Node 25	Cambisol	If B ≤ 14.59 and Ca (179.20; 189.20] and Mg ≤ 37.65 then Soil = Cambisol in 2.9% of cases
Node 26	Cambisol	If B ≤ 14.59 and Ca (179.20; 189.20] and Mg > 37.65 then Soil = Cambisol in 2.4% of cases
Node 27	Cambisol	If B ≤ 14.59 and Ca > 215.30 and Mn ≤ 2 then Soil = Cambisol in 7.1% of cases
Node 28	Cambisol	If B ≤ 14.59 and Ca > 215.30 and Mn > 2 then Soil = Cambisol in 1.2% of cases

## Data Availability

The original contributions presented in the study are included in the article and Supplementary Materials; further inquiries can be directed to the corresponding author.

## Supplementary Materials

The following supporting information can be downloaded at: https://www.mdpi.com/article/10.3390/foods13132006/s1, Table S1: Pollen profile of analyzed samples; Figure S1: Location of analyzed samples, Figure S2: CART Classification tree of soil types; Document S1: ICP-MS 7900 methods.
