# Supplementary material for: Exploring the Influence of Soil Types on the Mineral Profile of Honey: Implications for Geographical Origin Prediction
Source: Foods. 2024 Jun 25;13(13):2006. doi: 10.3390/foods13132006 (PMC11241210; doi:10.3390/foods13132006)
Supplement: Supplementary file 1 [file foods-13-02006-s001.zip › Document S1 ICP-MS 7900 methos.pdf]

Method for the determination of potentially toxic elements, metals and metalloids using the Agilet 7900 inductively coupled plasma mass detector.

The prepared samples are analyzed using an Agilent ICP-MS 7900 instrument by the method of mass spectrometry with inductively coupled plasma. The process operates as follows: the sample solution is nebulized and sprayed into a radiofrequency plasma, where a high-energy transfer process occurs instantly, resulting in a dissociation reaction that leads to atomization and ionization of the individual elements present in the sample.

The generated ions are extracted from the plasma in a vacuum chamber using built-in integrated ion optics and are subsequently directed into the mass spectrometer, which is based on a quadrupole. In this device, the ions are separated based on their mass-to-charge ratio. From the quadrupole separation unit, the ions pass directly into the detection unit, which is typically an electron multiplier. The data from the detection unit, corresponding to the quantity of ions present in the sample, are processed by computer software. Quantitative analysis is evaluated using calibration curves. The qualitative parameters of the methods are given in Table S1a.

Conditions for determination

RF Power:	1370 W
RF Matching:	1,58 V
Plasma Gas (Ar) Flow:	15 l/min
Sampling Depth:	7,9 mm
Carrier Gas (Ar) Flow:	1,22 l/min
Nebulizer Pump:	0,1 rps
Spray Chamber Temp:	2°C

Sample preparation

A 0.3 g portion of the homogenized sample was weighed. The sample was transferred into a Teflon vessel designated for mineralization. 5 ml of nitric acid and 1 ml of hydrogen peroxide were added. The Teflon vessel was then sealed and placed into the mineralization device. After mineralization, the sample solution was quantitatively transferred into a 25 ml volumetric flask and filled up with deionized water. This prepared sample was then used for analysis.

Table S1a: Qualitative parameters of the ICP-MS 7900 methods

	Range of determination	0.3 g (mg/kg)		Uncertainty (%)
	(mg/kg,mg/l)	LOD	LOQ	
**Al**	0,00046-500	0,01957	0,03915	18
**As**	0.00005-0.5	0.00231	0.00462	19
**B**	0.00042-500	0.01778	0.03556	20
**Ca**	0.00015-500	0.00639	0.01278	20
**Cr**	0.00015-0.5	0.00650	0.01301	20
**Cu**	0.00015-0.5	0.00663	0.01327	18
**Fe**	0.00010-500	0.00430	0.00861	18
**K**	0.00004-500	0.00182	0.00365	19
**Mg**	0.00010-500	0.00416	0.00833	19
**Mn**	0.00003-500	0.00161	0.00323	18
**Na**	0.00001-500	0.00070	0.00140	18
**Ni**	0.00020-0.5	0.00865	0.01731	18
**Pb**	0.00003-0.5	0.00135	0.00271	20
**Zn**	0.00022-500	0.00939	0.01878	19
