# Supplementary material for: Exploring the Influence of Soil Types on the Mineral Profile of Honey: Implications for Geographical Origin Prediction
Source: Foods. 2024 Jun 25;13(13):2006. doi: 10.3390/foods13132006 (PMC11241210; doi:10.3390/foods13132006)

Table S1: Pollen profile of analysed samples

Sample	GPS	Brassica sp.	Corylus sp.	Artemisia sp	Rosaceae sp.	Rubus sp.	Salix sp.	Acer sp.	Castanea sp.	Trifolium sp.	Tilia sp.	Phacelia sp.	Umbelliferae sp.	Vicia sp.	Myosotis sp.	Lotus sp.	Hypericum sp.	Minor pollen (≤ 3 %)
S1	50.239575; 12.989521	0	1	29	43	1	0	0	1	1	0	9	2	0	3			Taraxacum sp.
S2	50.239575; 12.989521	2	8	0	29	0	0	7	0	5	0	1	0	7	3			Achillea sp
S3	49.428654; 14.418156	65	0	2	0	0	0	0	9	0	0	2	0	0	1			Robinia sp.
S4	50.297663; 15.942283	53	0	4	22	0	0	1	1	0	0	5	2	1	0			Robinia sp., Bellis sp., Helianthum sp., Rhamnus sp., Taraxacum sp.
S5	50.297663; 15.942283	40	0	0	23	7	2	0	6	0	2	7	1	0	5			Bellis sp., Helianthum sp., Rhamnus sp.
S6	50.640664; 13.824446	67	0	0	2	0	3	0	0	1	0	4	2	10	7			
S7	50.640664; 13.824446	67	0	0	1	0	2	0	0	2	0	3	8	11	2			
S8	48.83022; 16.40204	7	0	0	1	1	4	1	0	38	1	6	9	2	16			Taraxacum sp., Helianthum sp.
S9	50.772556; 14.212761	14	0	0	5	1	2	5	0	24	2	9	3	8	18			
S10	49.838954; 14.674457	15	0	2	10	0	0	2	0	8	0	1	4	2	39			Taraxacum sp.
S11	50.378809; 15.468937	25	0	3	8	0	2	0	0	26	13	0	4	2	3			Robinia sp.
S12	49.193826; 16.645596	65	0	1	2	0	0	0	0	0	1	0	26	0	0	0	0	
S13	49.272624; 16.436833	10	0	4	6	0	0	0	0	1	25	8	8	10	10			
S14	49.862371; 18.015278	52	0	1	10	0	0	10	0	3	0	0	1	0	1	16	0	
S15	49.195331; 18.099708	4	0	1	15	0	0	13	0	14	4	0	4	7	8	9	0	Taraxacum sp., Alnus sp.
S16	48.890935; 17.313834	43	0	0	5	0	0	2	0	15	7	0	2	3	0	16	0	
S17	50.096027; 17.662048	6	0	0	10	0	0	6	0	40	0	0	11	4	1	5	0	Alnus sp., Taraxacum sp.
S18	49.65563; 17.28393	54	0	1	1	0	0	7	0	3	0	0	5	2	0	22	0	Robinia sp.
S19	50.180311; 13.691346	7	0	0	0	10	0	0	0	55	0	0	2	0	7			
S20	49.838162; 18.255804	0	0	2	6	0	0	0	4	34	10	0	0	1	36			Bellis sp.
S21	49.838162; 18.255804	17	0	0	1	0	1	0	0	58	11	3	2	1	0	3	0	Campanula sp.
S22	50.002290; 17.418489	17	0	0	6	0	1	0	0	22	6	7	4	2	19	4	1	Betula sp., Rhamnus sp., Lythrum sp., Campanula sp., Taraxacum sp.
S23	49.584866; 18.095474	0	0	0	0	1	0	0	0	30	39	1	1	0	11	1	0	Echium sp.
S24	49.574144; 18.020926	2	0	0	0	0	0	0	0	17	35	0	1	1	19	2	0	Bellis sp., Thymus sp.
S25	50.192131; 17.651339	3	0	0	0	0	1	0	0	6	2	0	13	19	7	2	0	Robinia sp., Thymus sp., Lythrum sp., Udatna sp., Campanula sp.
S26	50.228148; 17.679459	28	0	0	0	0	5	0	0	8	5	0	8	5	8	1	0	Robinia sp., Bellis sp., Thymus sp., Campanula sp.
S27	49.145178; 16.514745	4	0	0	0	0	0	0	0	23	2	8	7	4	38	2	1	Taraxacum sp., Udatna sp., Fagopyrum sp.
S28	49.001167; 15.575487	32	0	0	0	0	12	0	0	1	0	3	0	0	1	10	0	Robinia sp., Lythrum sp.
S29	50.356195; 15.921364	63	0	0	2	0	10	1	0	10	0	0	1	0	1	6	0	Echium sp., Lythrum sp., Picea sp.
S30	48.905919; 16.942303	31	0	0	1	0	5	0	0	22	3	0	4	3	0	0	5	Helianthum sp., Thymus sp., Echium sp., Lythrum sp., Udatna sp.
S31	49.124226; 17.691972	4	0	0	44	0	0	34	0	5	1	0	2	3	2	1	0	Taraxacum sp., Robinia sp., Impatiens sp., Udatna sp., Campanula sp.
S32	49.431955; 13.222743	51	0	0	21	0	5	0	0	4	1	0	7	3	1	2	0	Taraxacum sp., Betula sp., Echium sp., Campanula sp.

Location of analysed samples

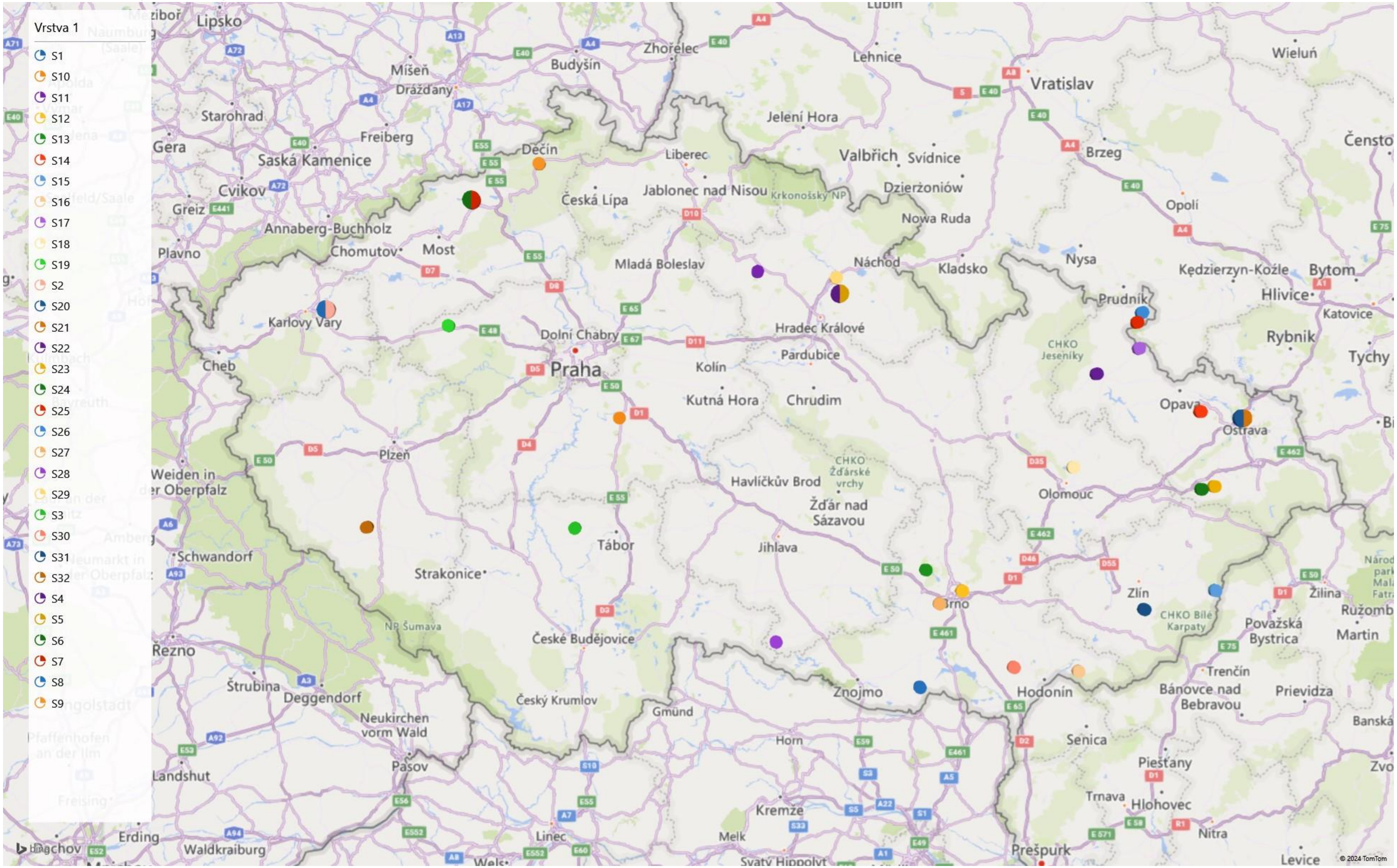

Supplement: Supplementary file 1 [file foods-13-02006-s001.zip › Table S1 Pollen profile.pdf]
