# Supplementary figures and images for: Exploring the Influence of Soil Types on the Mineral Profile of Honey: Implications for Geographical Origin Prediction
Source: Foods. 2024 Jun 25;13(13):2006. doi: 10.3390/foods13132006 (PMC11241210; doi:10.3390/foods13132006)

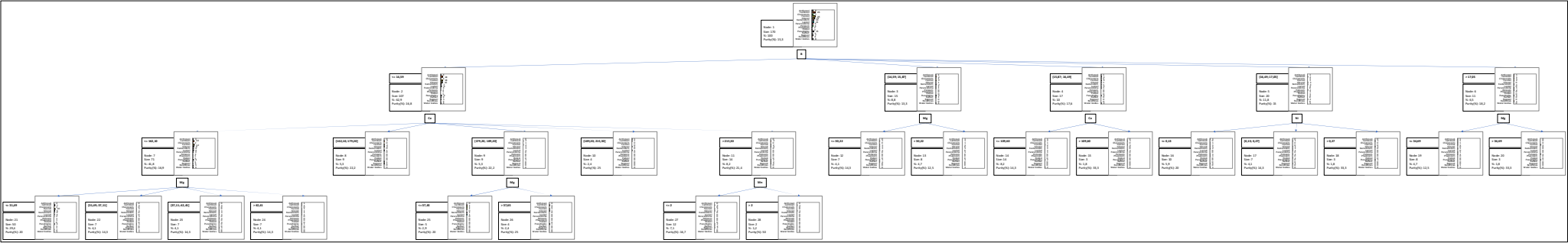

Supplement: Supplementary file 1 [file foods-13-02006-s001.zip › Figure S1 CART Classification tree.pdf]
